# Dietary constituents reduce lipid accumulation in murine C3H10 T1/2 adipocytes: A novel fluorescent method to quantify fat droplets

**DOI:** 10.1186/1743-7075-8-30

**Published:** 2011-05-12

**Authors:** Ines Warnke, Regina Goralczyk, Erna Fuhrer, Joseph Schwager

**Affiliations:** 1 DSM Nutritional Products Ltd.; Department of Human Nutrition and Health, CH-4002, Basel, Switzerland

## Abstract

**Background:**

Adipocyte volume (fat accumulation) and cell number (adipogenesis) is increased in obese individuals. Our objective was the identification of dietary constituents with inhibitory effects on triglyceride formation during adipogenesis. Therefore an *in vitro *adipose cell assay in murine C3H10 T1/2 cells was developed, which enabled rapid quantification of intracellular fat droplet accumulation during adipocyte differentiation. Results were corroborated by expression levels of several specific adipogenic and lipogenic genes which are known to regulate triglyceride accumulation.

**Methods:**

C3H10 T1/2 adipocyte differentiation was conducted with rosiglitazone in the presence of test compounds for 7 days. Accumulation of intracellular lipid droplets was measured using the Cellomics^® ^ArrayScan^® ^VTI HCS reader and SpotDetector^® ^BioApplication from ThermoFisher. Fluorescent images were automatically acquired and analysed employing the fluorescent dyes BODIPY^® ^493/503 and Hoechst 33342, for staining neutral lipids and localisation of nuclei, respectively. The expression levels of adipogenic and lipogenic genes, such as PPARα and PPARγ, C/EBPα, aP2, adiponectin, LPL and HSL, CPT-1β, ACC1, Glut4 and FAS, were determined by quantitative RT-PCR. Dietary ingredients including PUFAs, carotenoids, polyphenols and catechins were tested for their effect on lipid accumulation.

**Results:**

The ω-3 PUFAs docosahexaenoic acid (DHA) and eicosapentaenoic acid (EPA), the carotenoid β-carotene and hydroxytyrosol exhibited the strongest inhibitory effects on the rosiglitazone-stimulated lipid formation. (all-E)-lycopene and epigallocatechin gallate (EGCG) showed a moderate inhibition, whereas resveratrol did not reduce fat droplet formation. Additionally, it was demonstrated that adipogenic and lipogenic gene expression was attenuated. DHA, β-carotene and hydroxytyrosol inhibited the gene expression of PPARγ, C/EBPα, aP2 and CPT-1β.

**Conclusion:**

This *in vitro *assay in differentiating adipocytes enables automated detection and quantification of changes in lipid droplet number, size and intensity. The observed inhibitory effects of identified dietary constituents such as ω-3 PUFAs and β-carotene correlate with the modulation of genes involved in adipocyte differentiation.

## Background

The metabolic disorder obesity leads to various diseases such as hypertension, type-2-diabetes, respiratory complications and coronary heart disease [1]. This accounts for the numerous studies on cellular and molecular processes underlying fat metabolism in recent years [2,3].

Adipocytes are specialised cells that store triacylglycerides (TGs) in times of energy excess and release energy by lipolysis during energy shortage [2]. A constant positive energy balance leads to an excessive fat accumulation in white adipose tissue (WAT). Two mechanisms make this possible: (1) hypertrophy of adipocytes and (2) hyperplasia of proliferating pre-adipocytes into differentiated adipocytes [4]. This complex process called adipogenesis is sequentially regulated by several transcription factors such as peroxisome proliferator-activated receptor gamma (PPARγ) [5], CCAAT/enhancer binding proteins (C/EBPα, C/EBPβ and C/EBPδ) [6] and the adipocyte determination and differentiation factor 1 (ADD1/SREBP-1c) [3]. However, WAT is also an endocrine tissue that secretes metabolically active substances (adipokines), which function as feedback signals or lead to immunological responses [2].

The inhibition of differentiation of pre-adipocytes into adipocytes may regulate the amount of adipose tissue [7]. This has triggered the discovery of pharmacological inhibitors of adipogenesis and intensified the search for dietary ingredients with similar properties [8]. Food constituents such as polyphenols or carotenoids are dietary substances that are precursors of, or function as, signalling molecules. Most of these substances are plant-derived, being present in fruits, vegetables and nuts; likewise, polyunsaturated fatty acids (PUFAs) in fish and algae have similar properties. Many food ingredients have been described as modulators of adipocyte differentiation e.g. dietary PUFAs (for review see Madsen *et al*. [9]).

Several cell models exist to simulate differentiation of pre-adipocytes *in vitro*, the most widely-used being the 3T3-L1 cell line [10] derived from 3T3 cells [11]. Another commonly-employed model is the multipotent embryonic fibroblast cell line C3H10 T1/2 [12]. Pre-adipocyte differentiation is assessed through visualisation of accumulated fat droplets via neutral lipid staining. Alternatively, late adipocyte differentiation markers and genes related to lipid metabolism, such as lipoprotein lipase (LPL) [13], adipocyte fatty acid binding protein (aP2) [14], fatty acid synthase (FAS) [15], hormone-sensitive lipase (HSL) [16] or carnitine palmitoyltransferase 1 (CPT-1), can be measured.

The identification of bioactive compounds that might reduce excessive WAT requires valid *in vitro *test systems that allow for the investigation of a greater number of compounds and rapid quantitative detection of relevant fat cell differentiation features.

The main objective of the present investigation was to identify the effects of food constituents that could modulate the differentiation of C3H10 T1/2 cells into mature adipocytes and the concomitant accumulation of cytosolic TGs. For this purpose we have developed a new morphological, high content, cell assay (HCA) using the Cellomics^® ^ArrayScan^® ^VTI HCS Reader and the SpotDetector^® ^BioApplication software from ThermoFisher. Several chemical classes of dietary ingredients, such as PUFAs, carotenoids, polyphenols and catechins, were tested in this assay. Furthermore we examined the effects of these compounds on expression levels of genes known to play key roles in adipocyte differentiation and fat metabolism. Our data demonstrate that the HCA assay is a valuable substitute for the commonly-used Oil Red O procedure [17], enabling improved *in situ *quantification of fat droplet number, size and intensity in adipocytes.

## Methods

### Materials

Dulbecco's modified Eagle's cell culture medium (DMEM), penicillin G (10000 units/ml) and streptomycin (10000 μg/ml) solution (pen/strep), L-glutamine (100×, 200 mM) and phosphate-buffered saline without Ca^2+ ^and Mg^2+ ^(PBS (-/-)) were from GIBCO™ (Grand Island, NY, USA). Fetal calf serum (FCS) and trypsin were purchased from AMIMED^® ^(BioConcept, Allschwil, Switzerland). FALCON^® ^culture flasks were from BECTON DICKINSON (Franklin Lakes, NJ, USA) and 24-well plates coated with collagen type I were supplied by Greiner bio-one (Frickenhausen, Germany).

Rosiglitazone was purchased from Shanco International (NJ, USA) and recombinant bovine insulin (dissolved in H_2_O), resveratrol and ω-3 PUFAs, docosahexaenoic acid (DHA) and eicosapentaenoic acid (EPA), were from Sigma-Aldrich (St. Louis, MO, USA). 3-hydroxytyrosol (HT) was from Cayman Chemicals (Ann Abor, MI, USA). Epigallocatechin gallate (EGCG, Teavigo™) and carotenoids were supplied by DSM Nutritional Products Ltd (Basel, Switzerland). Carotenoids were dissolved in tetrahydrofurane (THF). All other test compounds were dissolved in dimethylsulfoxide (DMSO).

Fluorescent dyes - Hoechst 33342 and BODIPY^® ^493/503 - were obtained from Molecular Probes™ (Eugene, OR, USA). The CytoTox^® ^non-radioactive cytotoxicity assay kit was purchased from Promega (Fitchburg, WI, USA). Primers and probes were synthesised by Applied Biosystems (PE Biosystems, Foster City, CA, USA) or by Sigma Genosys (St. Louis, MO, USA).

### Cell culture

Mouse embryonic fibroblast cells C3H10 T1/2 [12] were from ATCC-LGC (Middlesex, UK) and cultured according to the supplier's protocol. Briefly, cells were grown in high glucose (4.5 g/ml) DMEM supplemented with 10% FCS and 0.5% pen/strep (v/v) (growth medium; GM) at 37°C and 5% CO_2_. Cells were cultured until they reached 80 - 90% confluence; they were used between passage 6 and 20 for the experiments described below.

### Adipocyte differentiation assay

C3H10 T1/2 cells were plated (2 × 10^5 ^cells/cm^2^) in collagen-I-coated 24-well plates and maintained in phenol red-free GM supplemented with 2 mM L-glutamine (GM1) until 1 day post-confluence (day 0). Differentiation medium (DM) containing 200 nM bovine insulin and 10 μM rosiglitazone was then added [18]. C3H10 T1/2 pre-adipocytes were either cultured in DM only (positive control) or in DM supplemented with different doses of test compounds (0.5 - 25 μM) for 4 days (gene expression study) or 7 days. Each treatment was performed in triplicate. The final DMSO and/or THF concentration was adjusted for all treatments and controls to 0.2% and/or 0.1%, respectively, and medium and compounds were renewed every other day. Cells were lysed after 96 h or, after 1 week treatment, fixed with 60% isopropanol for 1.5 h at 4°C and stained (see below).

### Staining procedures

Adipocytes were stained with Oil Red O or with the fluorescent dyes Hoechst 33342 (Hoe) and BODIPY^® ^493/503 (BP). In brief, fixed cells were first washed twice with PBS (-/-). For the Oil Red O staining, cells were incubated with 200 μl of Oil Red O solution (0.33% w/v in 60% isopropanol) for 20 min at room temperature (RT). The fluorescent staining comprised addition of 200 μl Hoe/BP solution (3 μg/ml Hoe, 0.75 μg/ml BP in PBS (-/-)) per well, followed by incubation in the dark at RT for 30 min. Before analysis, the cells were washed twice with PBS (-/-).

### Visualisation of Oil Red O stained adipocytes and quantification of Oil Red O

Images of Oil Red O stained adipocytes were acquired using a Nikon Coolpix 990 camera at 20× magnification and further edited with Adobe^® ^Photoshop^® ^CS2 version 9.

To quantify staining of fat droplets, Oil Red O stain was extracted by adding isopropanol to each well immediately after removal of the wash solution (300 μl per well). Plates were incubated for 10 min at RT; two aliquots of 100 μl were transferred to 96-well plates and read immediately at 510 nm (Spectra Max Plus 384, Molecular Devices, CA USA). After subtraction of the background value (wells without cells, but treated with medium; non-specific binding), the differentiation of treated cells was calculated as percent of positive control.

### Automated determination of lipid accumulation in adipocytes

Lipid droplets were quantified with the Cellomics^® ^ArrayScan^® ^VTI HCS Reader (500 series, version 5.6.1.4 - 0.63×, ThermoFisher Scientific). Fat droplets were detected with the provided SpotDetector^® ^BioApplication. The fluorescent dye, BODIPY^® ^493/503, was used to visualise and quantify fat droplets (number, area, intensity) in differentiated C3H10 T1/2 adipocytes, while nuclei were stained using the Hoechst 33342 dye. The method is based on a two-channel assay, which uses a 40× objective (NA 0.5), a Hamamatsu ORCA-ER digital camera in combination with a 0.63× coupler and Carl Zeiss microscope optics for image acquisition. Images were acquired in high resolution (1024 × 1024, 1 × 1) and auto focus mode (1024 × 1024, 4 × 4) resulting in a field width of 262 microns. Channel one (Ch1) applies the XF93-Hoechst filter and is the focus channel in which objects (nuclei) are identified, and the spots (fat droplets) are detected in channel two (Ch2, XF93-FITC filter).

The default settings of the SpotDetector.V2 assay algorithm version 4.1 were altered such that, in Ch2, thresholds were set to ensure that only fat droplets of a certain size and intensity were selected for analysis of 100 fields per well (Table 1). The data of each channel were reported on a "per field" basis.

**Table 1:** Protocol parameters differing from the standard SpotDetector^® ^algorithm

Object Identification			
Channel 1	Method:	FixedThreshold	
	Value:	125	
Channel 2	Method:	TriangThreshold	
	Value:	0.499	
Object Selection Parameter		Min	Max
**Channel 1**	ObjectArea:	100	6000
**Channel 2**	SpotAreaCh2:	3	10000
	SpotAvgIntenCh2:	30	4095
	SpotTotalIntenCh2:	1691	4411431044

Assay Parameters			
	BackgroundCorrection:	140	
	ObjectSegmentationCh1:	20	
	SmoothFactorCh1:	5	
	SpotDetectRadiusCh2:	15	
	TargetCircModifierCh2:	512	
	PixelSize:	0.256 microns	

The alteration of assay parameters enables adjustment of the algorithm to different biological cell models. Displayed values are pixel number.

### Cell viability

Cell viability was assessed using the CytoTox^® ^non-radioactive cytotoxicity assay kit (lactate dehydrogenase (LDH) determination) and by calculating the relative cell number according to the following equation: cell number (%) = (average number of cells per fields [treatment] * 100/average number of cells per fields [control]). Cytotoxic effects of the compounds were rated by comparison of the LDH levels in the supernatants of compound-treated and untreated cells at day 7. Nuclei were counted with the ArrayScan^® ^reader.

### Gene expression study

Total mRNA was extracted using the RNeasy^® ^96 Kit (Qiagen, Hilden, Germany) and quantified with the RiboGreen^® ^Kit (Invitrogen, Molecular Probes™, Eugene, OR, USA) according to the manufacturer's protocols. RNA quality was assessed using RNA 6000 Nano Chips for the Agilent 2100 Bioanalyzer (Agilent Technologies, USA). For first strand cDNA synthesis 650 ng total RNA was reverse-transcribed using the Omniscript^® ^RT Kit from Qiagen (20 μl reaction mix), random primers from Promega and RNaseOUT™ from Invitrogen. After three incubation steps (60 min at 37°C, 5 min at 93°C, 5 min at 4°C), cDNA solutions were diluted with DEPC-treated water to 260 μl and stored at -20°C.

Quantitative TaqMan™ RT-PCR was performed using an ABI-PRISM^® ^7900 HT Sequence Detection System (PE Biosystems, Foster City, CA, USA) and MicroAmp^® ^Optical 96-well reaction plates (PE Biosystems, Foster City, CA, USA). Briefly, 5 μl cDNA was added to 20 μl reaction mixture, containing 1 × Universal Master Mix (PE Biosystems, Rotkreuz, Switzerland), 300 nM PCR primers (forward and reverse), and 100 nM TaqMan™ probe (FAM-TAMRA) for the gene of interest. 18S rRNA was used as endogenous control (EC), with primers and probes (VIC-TAMRA) at 50 nM and 100 nM, respectively. Primers and probes were designed using the Primer Express software (Applied Biosystems, Forster City, CA, USA). The oligonucleotide sequences for the primers and probes are shown in Table 2. The cycle conditions were: 2 min at 50°C, 10 min at 95°C, 40 cycles of 15 sec at 95°C and 60 sec at 60°C. Threshold C_T _values were set at 0.05. Baseline start and stop values for the gene of interest were set at 3 and 15, respectively, and for 18S rRNA at 3 and 7, respectively. mRNA abundance was calculated using the comparative C_T _method according to the manufacturer's protocol. Shortly, ΔC_T _= C_T _[*gene of interest*] - C_T _[*EC*] and ΔΔC_T _= ΔC_T _[*rosiglitazone control cells*] - ΔC_T _[*treated cells*]. The fold expression for the gene of interest was expressed as .

**Table 2:** Sequences of primers and probes for different adipocyte specific genes used in real-time RT-PCR

Gene name	Forward/Reverse Primer	Probe
**Adiponectin (Acrp30)**	5'-GGCACTCCTGGAGAGAAGGG 5'-ATTCCAACATCTCCTGTCTCACC	5'-GAAAGGAGATGCAGGTCTTCTTGGTCCTA

**Fatty acid binding protein 4, adipocyte type (aP2)**	5'-GCGTGGAATTCGATGAAATCA 5'-CCCGCCATCTAGGGTTATGA	5'-CGCAGACGACAGGAAGGTGAAGAGC

**Acetyl-CoA carboxylase (ACC1)**	5'-TCACTCGCTTTGGAGGCAA 5'-CGCAGCGATGCCATTGT	5'-AGGGTCATAGAGAAGGTGCTCATCGCC

**Lipoprotein lipase (LPL)**	5'-GTGGCCGAGAGCGAGAAC 5'-AAGAAGGAGTAGGTTTTATTTGTGGAA	5'-TTCCCTTCACCCTGCCCGAGG

**Hormone sensitive lipase (HSL)**	5'-AAGACCACATCGCCCACAG 5'-CAGACACACTCCTGCGCATAG	5'-AGAGTCTGTGCGCCCCACGGA

**Fatty acid synthase (FAS)**	5'-TCATAAAGCAGTTTCTTGATGTGGA 5'-CAGGCTCTTCAGTGGCAGC	5'-CACAGCAAGGTGCTGGAGGCCC

**Carnitine palmitoyl transferase β (CPT-1 β)**	5'-CCAATCATCTGGGTGCTGG 5'-TAAGAGACCCCGTAGCCATCAT	5'-TGGCTTTGGTCCCGTGGCG

**Glucose transporter 4 (Glut4)**	5'-TCGGCTCTGACGATGGG 5'-CCAAGCCAGCTGAGAATACAG	5'-AACCCCCTCGGCAGCGAGTGACTG

**Peroxisome proliferator activated receptor gamma 2a (PPARγ 2a)**	5'-CTATGAGCACTTCACAAGAAATTACCAT 5'-TCCATCACGGAGAGGTCCAC	5'- TCTGGCCCACCAACTTCGGAATCAG

**Peroxisome proliferator activated receptor alpha (PPARα)**	5'-GCCTCAGGGTACCACTACGG 5'-GCCGAATAGTTCGCCGAA	5'-CACGCATGTGAAGGCTGTAAGGGCTT

**CCAAT/enhancer binding protein alpha (C/EBPα)**	5'-CGGTGCGGGCAAAGC 5'-TGCGTTCCCGCCGTAC	5'-AGAAGTCGGTGGACAAGAACAGCAAC

**18S rRNA**	5'-CGGCTACCACATCCAAGG 5'-CGGGTCGGGAGTGGGT	5'-TTGCGCGCCTGCTGCCT

### Statistical analysis

Statistical significance of the mean differences between treatment and positive control was tested by Student's t-test for unpaired values. P values less than 0.05 were considered significant. ArrayScan^® ^results are shown as mean ± SEM. Gene expression data are expressed as fold change ± error (based on SEM).

## Results

### Cross-validation of Cellomics^® ^assay with Oil Red O method

For validation of the Cellomics^® ^assay, the established Oil Red O method was used as reference, and the accumulation of intracellular lipid droplets was measured with both methods. C3H10 T1/2 cells were differentiated for 7 days using the PPARγ agonist rosiglitazone, at concentrations between 0.01 and 100 μM. Cells grown in DM without rosiglitazone (negative control) accumulated only a few small fat droplets. The average number of fat droplets per cell increased from 0.5 (negative control) to 4.0 (positive control). The fat droplets were both significantly brighter and larger (p < 0.001) in cells incubated with 10 μM rosiglitazone.

To assess the quality and suitability of the new methodology the assay windows (positive control (p)/negative control (n)) and the estimated Z-factors (1- ((3 × (σ_p _+ σ_n_))/(| μ_p _- μ_n _|); σ = sample means and σ = sample standard deviation) for the different assay parameters were determined. A Z-value between 0.5 and 1 is interpreted as an exceptional assay, whereas a value between 0 and 0.5 is indicative of a marginal assay [19]. The Oil Red O method had an assay window of 2.3 and a Z-factor of only -0.08; where 0.2% DMSO was used as negative control and 100 μM rosiglitazone as maximum positive control. In contrast, the Cellomics^® ^assay resulted in a window of 6.8 and a Z-factor of 0.71 for the Spot Count/Object parameter. During screening, an assay window greater than 20 and an average Z-value of 0.65 was achieved with 10 μM rosiglitazone as positive control and 0.2% DMSO as negative control, indicating that the fluorescent method is a robust assay (CV Spot Count/Object = 0.09) [19].

**Figure 1:**
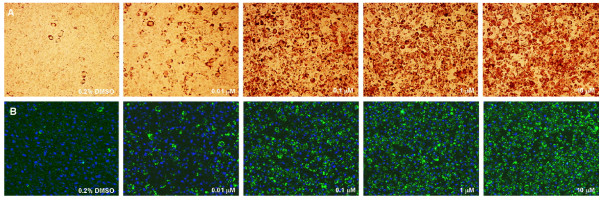
**Visualisation of lipid accumulation in differentiated adipocytes**. C3H10 T1/2 cells were treated for 7 days with rosiglitazone (0.01, 0.1, 1 and 10 μM) and, after fixation with 60% isopropanol, stained with A) Oil Red O or B) Hoechst 33342 and BODIPY^® ^493/503. Images were acquired using A) a Nikon Coolpix 990 camera at 20× magnification or B) the Cellomics^® ^HCS Reader camera (20×).

**Figure 2:**
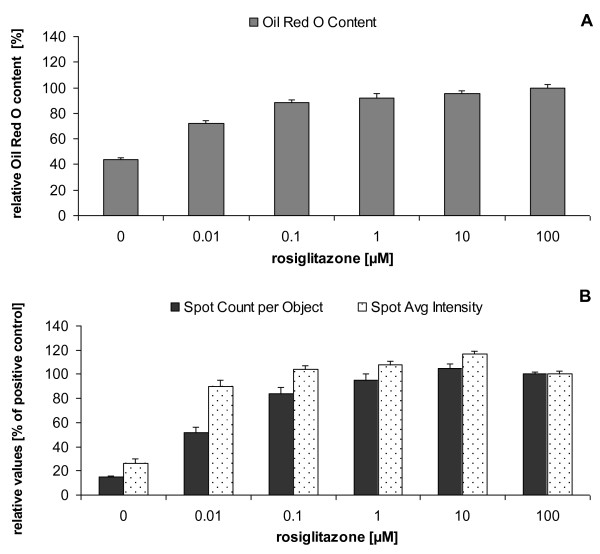
**Determination of intracellular fat content in adipocytes differentiated for 7 days with varying concentrations of rosiglitazone**. (A) Oil Red O quantification according to the Materials and Methods section. (B) Fat droplet number (Spot Count/Object) and droplet intensity (Spot Avg Intensity), using the Cellomics HCS Reader. Neutral lipids were stained with BODIPY^® ^493/503. Results are expressed as percentage of values obtained with 100 μM rosiglitazone (mean ± SEM, n = 8).

Rosiglitazone dose-dependently increased the number of Oil Red O-stained lipid droplets (Figure 1A). To quantify the microscopic findings, Oil Red O was eluted from the fat droplets and measured spectro-photometrically (Figure 2A). A differentiation plateau seemed to be reached between 10 μM and 100 μM rosiglitazone, hence 100 μM values were used as references for calculations. The fluorescent stain BODIPY^® ^likewise enabled measurement of rosiglitazone-dependent induction of TG formation (Figure 1B). Both fat droplet number (Spot Count/Object) and fat droplet intensity (Spot Avg Intensity) concentration-dependently increased with rosiglitazone (Figure 2B). Thus, our method proved valid for detecting intracellular lipid accumulation.

### Effects of substances on number of maturing C3H10 T1/2 cells

Cell (or nuclei) number was considered to reflect cell viability. Cells treated with different concentrations of DHA, EPA, EGCG, HT, resveratrol, (all-E)-lycopene and β-carotene had a similar nuclei count as control cells. The number of cells was slightly reduced (< 20%) by incubation with EGCG, HT, resveratrol and β-carotene at the highest concentration (data not shown). This was corroborated by the measurement of LDH activity in supernatants after 7 days' treatment. Only DHA and EPA increased the LDH release at 25 μM (data not shown). The data indicate that cell viability was not impaired by the substances, although some of them might have apoptotic effects at high concentrations.

### PUFAs and β-carotene inhibit lipid accretion in maturing adipocytes

**Figure 3:**
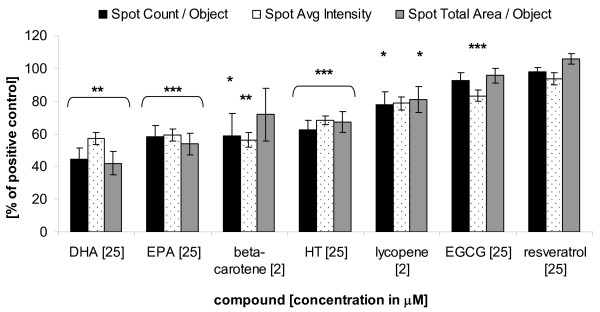
**Effects of DHA, EPA, β-carotene, HT, (all-E)-lycopene, EGCG and resveratrol on lipid formation measured with the HCA assay**. C3H10 T1/2 cells were treated for 7 days. Shown are three parameters (Spot Count/Object, Spot Avg Intensity and Spot Total Area/Object) that quantify lipid formation, as compared to rosiglitazone controls set as 100%. Data are shown as mean ± SEM (n = 10 - 20). Student's t-test: treatment versus control (*) *p *< 0.05, (**) *p *< 0.01, (***) *p *< 0.001.

Subsequently, the effects of test substances on lipid accumulation in adipocytes were investigated using the Cellomics^® ^method. Murine pre-adipocytes were differentiated for 7 days (n = 10) in the absence or presence of ω-3 PUFAs (DHA or EPA), carotenoids, (poly)-phenols, and catechins. Incubation of rosiglitazone-treated C3H10 T1/2 cells with 25 μM DHA, 25 μM EPA, or 2 μM β-carotene, significantly decreased the number of lipid droplets (Spot Count/Object) by 56%, 42% and 41%, respectively (Figure 3). At 25 μM HT inhibited the accumulation of TGs in adipocytes by 38%, whereas (all-E)-lycopene and EGCG reduced the fat droplets by 22 and 7%, respectively. The test compounds also lowered the spot intensity (Spot Avg Intensity) and Spot Total Area/Object parameters to a similar extent, suggesting that the total fat content per cell was reduced. Compared to the PUFAs, resveratrol did not affect adipogenesis and even increased TG content.

### Concentration-dependent effects of test compounds on TG accumulation

**Figure 4:**
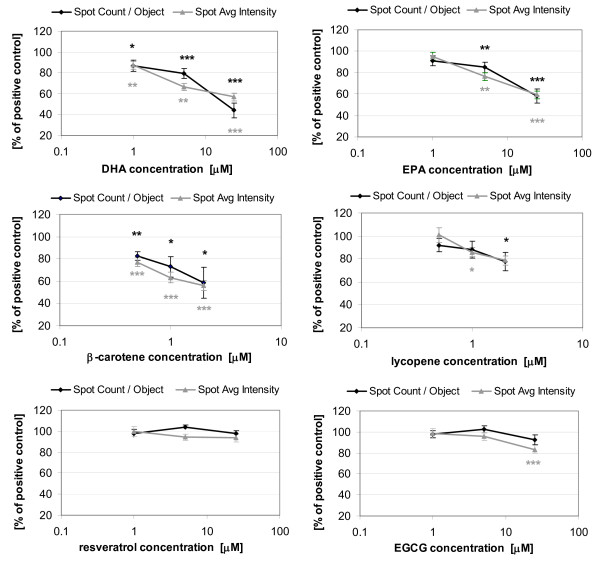
**Effects of varying concentrations of DHA, EPA, β-carotene, (all-E)-lycopene, resveratrol and EGCG on number and intensity of fat droplets**. C3H10 T1/2 cells were differentiated in the presence of test substances for 7 days. Spot Count/Object and Spot Avg Intensity were determined, as compared to rosiglitazone control set as 100%. Data are shown as mean ± SEM (n = 10 - 20). Student's t-test: treatment versus control (*) *p *< 0.05, (**) *p *< 0.01, (***) *p *< 0.001.

In order to investigate the concentration-response relationship of the test substances, we differentiated pre-adipocytes for 7 days in the presence of 3 concentrations of each test compound. DHA and EPA concentration-dependently reduced both fat droplet number and intensity (Figure 4). β-carotene showed a more potent concentration-dependency than (all-E-)-lycopene. EGCG and resveratrol, however, had no inhibitory effect on intracellular TG accumulation (except EGCG at 25 μM; Figure 3). In contrast, HT showed a similar concentration-dependent modification of fat droplets as EPA (data not shown).

### Reduced expression of genes involved in adipocyte differentiation and in glucose and fatty acid metabolism

To explore a possible impact of the tested substances at the transcriptional level, we differentiated pre-adipocytes with 10 μM rosiglitazone in the absence or presence of DHA, EPA, EGCG, HT, resveratrol, (all-E-)-lycopene or β-carotene and assessed gene expression levels by quantitative RT-PCR. Tested genes were the master regulators of adipogenesis PPARγ and C/EBPα, the nuclear receptor PPARα, as well as the adipocyte differentiation markers fatty acid binding protein 2 (aP2) and adipose tissue-specific adiponectin. mRNA levels of genes involved in fatty acid metabolism (ACC1, CPT-1β, FAS, HSL, LPL) and glucose uptake (Glut4) were also determined.

**Figure 5:**
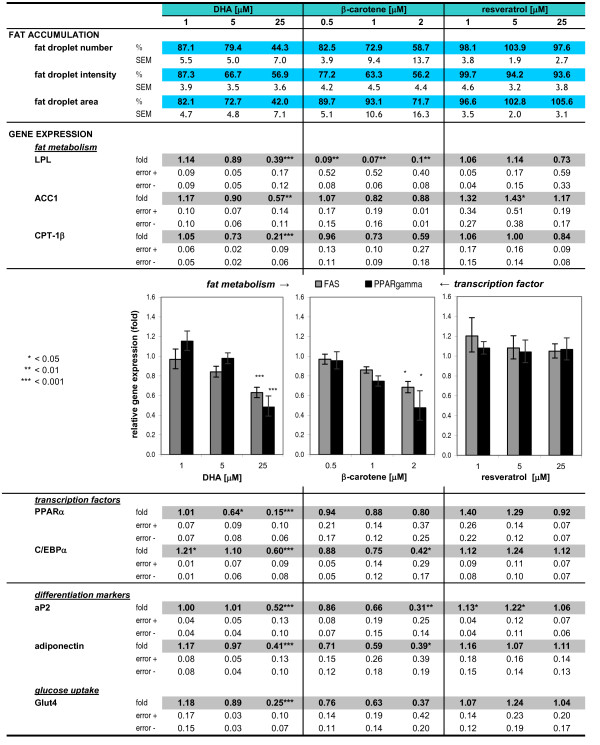
**Summary of overall effects of the dietary ingredients DHA, β-carotene and resveratrol on lipid accumulation and on gene expression clusters**. mRNA levels of enzymes involved in glucose and fat metabolism (Glut4, LPL, FAS, ACC1 and CPT-1β) and of adipocyte differentiation markers (PPARγ, C/EBPα, PPARα, aP2 and adiponectin) were determined in maturing C3H10 T1/2 cells. Depicted are the dose-dependent effects of DHA and β-carotene and the impact of resveratrol on lipid accumulation (after 7 days treatment, top of the table) and on gene expression (after 4 days treatment) relative to rosiglitazone control cells. Data are shown as % of positive control ± SEM (fat accumulation parameters) and fold change ± error (based on SEM, n = 6 - 15) for gene expression levels, respectively. Exemplarily, the fold changes of the genes FAS and PPARγ are shown as illustration. Fat droplet number equates to Spot Count/Object; fat droplet intensity equates to Spot Avg Intensity and fat droplet area equates to Spot Total Area/Object. Student's t-test: treatment versus control (*) p < 0.05, (**) p < 0.01, (***) p < 0.001. The average coefficient of variation (CV) for dCT values was less than 5% for all analysed genes at all concentration of the investigated compounds.

Treatment with DHA (25 μM) reduced the expression of aP2 and adiponectin, to 48% and 59% respectively (Table 3). The expression of aP2 and adiponectin after treatment with 2 μM β-carotene was also significantly decreased to 31% and 39%, and at 25 μM HT to 47% and 69%, respectively (Figure 5, Table 3). The mRNA levels of the adipogenesis-associated transcription factors, PPARγ, PPARα and C/EBPα were strongly suppressed by PUFAs, β-carotene and HT at the highest concentration (up to 85%; Table 3). Although PPARγ and C/EBPα were suppressed by β-carotene, as by DHA and EPA, not exactly the same down-stream genes were affected. Interestingly 1 μM DHA led to a significant increase in the mRNA level of C/EBPα.

**Table 3:** Effects of compounds on gene expression of adipocyte differentiation markers and enzymes involved in fat and glucose metabolism (n = 6 - 15)

		PUFAs		CAROTENOIDS		PHENOLS		
		DHA 25 μM	EPA 25 μM	lycopene 2 μM	β-carotene 2 μM	EGCG 25 μM	HT 25 μM	Resv 25 μM
**PPARγ2**	**fold**	**0.48**	**0.61**	**0.93**	**0.48**	**0.97**	**0.53**	**1.07**
	error +/-	0.11/0.09	0.09/0.08	0.33/0.25	0.17/0.13	0.20/0.17	0.15/0.12	0.12/0.11
	*p-value*	*< 0.001*	*0.002*	*0.75*	*0.04*	*0.64*	*< 0.001*	*0.53*

**PPARα**	**fold**	**0.15**	**0.25**	**1.01**	**0.80**	**0.81**	**0.47**	**0.92**
	error +/-	0.10/0.06	0.10/0.07	0.57/0.36	0.37/0.25	0.11/0.09	0.15/0.12	0.07/0.07
	*p-value*	*< 0.001*	*< 0.001*	*0.94*	*0.69*	*0.33*	*< 0.001*	*0.99*

**C/EBPα**	**fold**	**0.60**	**0.82**	**0.86**	**0.42**	**1.02**	**0.46**	**1.12**
	error +/-	0.09/0.08	0.08/0.07	0.34/0.24	0.29/0.17	0.13/0.12	0.15/0.11	0.07/0.07
	*p-value*	*< 0.001*	*0.038*	*0.12*	*0.02*	*0.94*	*< 0.001*	*0.44*

**aP2**	**fold**	**0.52**	**0.72**	**1.09**	**0.31**	**0.82**	**0.47**	**1.06**
	error +/-	0.13/0.11	0.05/0.05	0.13/0.12	0.25/0.14	0.07/0.07	0.15/0.11	0.07/0.06
	*p-value*	*< 0.001*	*0.005*	*0.26*	*0.007*	*0.04*	*< 0.001*	*0.45*

**Acrp30**	**fold**	**0.41**	**0.61**	**0.85**	**0.39**	**0.84**	**0.69**	**1.11**
	error +/-	0.13/0.10	0.12/0.10	0.11/0.10	0.39/0.19	0.09/0.08	0.08/0.07	0.14/0.13
	*p-value*	*< 0.001*	*0.002*	*0.06*	*0.02*	*0.79*	*< 0.001*	*0.11*

**Glut4**	**fold**	**0.25**	**0.39**	**1.33**	**0.37**	**0.86**	**0.42**	**1.04**
	error +/-	0.10/0.07	0.09/0.08	0.21/0.18	0.42/0.20	0.17/0.15	0.07/0.06	0.20/0.17
	*p-value*	*< 0.001*	*< 0.001*	*0.04*	*0.06*	*0.33*	*< 0.001*	*0.47*

**LPL**	**fold**	**0.39**	**0.62**	**0.41**	**0.10**	**0.63**	**0.77**	**0.73**
	error +/-	0.17/0.12	0.13/0.10	0.35/0.19	0.40/0.08	0.28/0.19	0.12/0.11	0.59/0.33
	*p-value*	*< 0.001*	*0.013*	*0.09*	*0.005*	*0.01*	*0.13*	*0.18*

**FAS**	**fold**	**0.63**	**0.65**	**0.79**	**0.68**	**1.13**	**0.83**	**1.05**
	error +/-	0.05/0.05	0.05/0.05	0.19/0.15	0.06/0.06	0.19/0.17	0.07/0.07	0.07/0.07
	*p-value*	*< 0.001*	*0.02*	*0.14*	*0.04*	*0.37*	*0.08*	*0.37*

**HSL**	**fold**	**0.58**	**0.75**	**0.91**	**0.49**	**1.04**	**0.69**	**1.05**
	error +/-	0.11/0.09	0.11/0.10	0.06/0.06	0.28/0.18	0.10/0.09	0.09/0.08	0.07/0.07
	*p-value*	*< 0.001*	*0.016*	*0.58*	*0.04*	*0.69*	*< 0.001*	*0.30*

**CPT-1β**	**fold**	**0.21**	**0.34**	**1.11**	**0.59**	**0.81**	**0.44**	**0.84**
	error +/-	0.09/0.06	0.11/0.08	0.28/0.22	0.27/0.18	0.10/0.09	0.06/0.05	0.09/0.08
	*p-value*	*< 0.001*	*< 0.001*	*0.23*	*0.35*	*0.08*	*< 0.001*	*0.47*

**ACC1**	**fold**	**0.57**	**0.64**	**0.90**	**0.88**	**1.46**	**0.68**	**1.17**
	error +/-	0.14/0.11	0.14/0.11	0.30/0.23	0.01/0.01	0.34/0.27	0.15/0.13	0.19/0.17
	*p-value*	*0.004*	*0.02*	*0.58*	*0.72*	*0.09*	*0.02*	*0.45*

ACC1, CPT-1β, FAS, HSL and LPL are enzymes which control triglyceride transport, fatty acid synthesis and transport of long-chain fatty acids in differentiated fat cells. In the case of CPT-1β, its expression was significantly reduced by DHA to 21% and by HT to 44%, but not by lycopene (Table 3). The effects of DHA and β-carotene on gene expression levels were concentration-dependent (Figure 5). LPL mRNA levels were strongly diminished to 10% by all β-carotene concentrations.

With regard to the glucose transporter 4 gene (Glut4), which is instrumental for adipocyte glucose uptake, the investigated substances had an effect that was similar to that observed for ACC1 (Figure 5). In particular, DHA affected the gene expression of Glut4 in a dose-dependent manner. Conversely, resveratrol slightly increased mRNA levels of the investigated genes (Figure 5). This is consistent with the effects on cell morphology described above.

## Discussion

The present study describes the ability of natural substances to modulate intracellular lipid accumulation during adipogenesis. A high-content method for detecting fat droplets in differentiating adipocytes is established. Lipid droplets are easily accessible for microscopic analysis, because the nuclei and the droplets can be well visualised in separate fluorescent channels. As a model system we selected a murine pluripotent cell line, C3H10 T1/2, which could be readily differentiated into adipocytes by treating the cells with the PPARγ agonist rosiglitazone and insulin.

The SpotDetector^® ^protocol from ThermoFisher was modified to identify fat droplets with a radius of > 15 pixel (i.e. > 3.9 μm), therefore excluding small droplets that are formed in pre-adipocytes only exposed to DMSO and insulin. The adapted algorithm quantified the effects of 10 μM rosiglitazone with high consistency, yielding Z-factors > 0.6 for the nuclei-related parameters Spot Count and Spot Area and for the parameter Spot Average Intensity. Hence the Cellomics^® ^assay is suitable for rapid screening of a large number of compounds [19] and is amendable for a high throughput screen if transferred to a 96-well-plate format. The consistent measurements achieved with the SpotDetector^® ^BioApplication also enabled detection of moderate effects of less potent modulators such as bioactive components in food. This high-content analysis (HCA) approach is a greatly improved method (higher Z-factors, broader window and small CVs) compared with the commonly-used Oil Red O procedure, delivering fast, multi-parametric and objective data in a labour-reduced manner. Its use is comparable with the system presented by McDonough and co-workers [20], who applied the CyteSeer^® ^image analysis software (Vala Sciences), together with a robotic microscopy instrument. However, the convenient use of commercially available cell culture plates, the easily applicable lipid stain (BODIPY^® ^493/503) and rapid field-based analysis of images with the provided BioApplication from ThermoFisher are advantageous for the fast quantification of fat droplets in adipocytes.

A large number of studies suggest that certain plant extracts, their constituent phytochemicals [8,21] and the fatty acid composition of fats [22], influence the metabolism in adipocytes [23]. Based on this information we tested several bioactive substances present in our daily diet with the established HCA assay.

The ω-3 PUFAs EPA and DHA, which are known to reduce lipid droplet size in differentiating 3T3-L1 pre-adipocytes [9,24] and to decrease adipose growth in rodents [25-28], were tested as reference compounds in our assay. ω-3 PUFAs markedly reduced the number of fat droplets and their average intensity in a dose-dependent manner whereas size of the lipid droplets was not decreased (data not shown). By comparison, Madsen *et al*. [9] used a mix of dexamethasone, 3-isobutyl-1-methylxanthine (IBMX) and insulin for the differentiation of 3T3-L1 cells and compared whether different PUFAs enhanced adipocyte differentiation to a similar extent as the PPARγ agonist rosiglitazone. DHA and EPA were reported to be less potent than rosiglitazone. Because we used only rosiglitazone and insulin for differentiation, one could expect that the concomitant treatment with DHA or EPA would further stimulate the TG accretion. Interestingly in maturing C3H10 T1/2 cells we observed the opposite effect. At which stages of the adipocyte life cycle (determination, clonal expansion, maturation, lipolysis or apoptosis) [2] the ω-3 PUFAs affect the reduction of lipid droplets in C3H10 T1/2 could not be deduced from the ArrayScan^® ^results. The elevated LDH levels after 7 days' treatment might indicate that DHA induced apoptosis during clonal expansion as described for 3T3-L1 cells [29]. Thus, the potent inhibition of lipid accumulation is not only due to decreased adipogenesis but might also reflect a pro-apoptotic effect of DHA.

In addition, we showed that EPA and DHA reduced expression levels of adipogenic and lipogenic genes as described by Raclot *et al*. [30] and Shillabeer *et al*. [31]. C/EBPα and PPARγ are the 'master' regulators of adipogenesis [32]. PPARγ regulates the anabolic arm of lipid metabolism [33], whereas PPARα [5,34] and PPARβ/δ are effective as catabolic modulators of the energy balance [35]. PPARγ and PPARα are induced in the early and late phases of the differentiation process, respectively [36,33], and act as sensors for fatty acids and their derivatives [37,38]. Our findings that C3H10 T1/2 adipocytes, differentiated in the presence of DHA or EPA, show only a weak C/EBPα and PPARγ expression, are in line with the results of Worgall *et al*.: PUFAs down-regulated the expression of sterol regulatory element-binding proteins (SREBPs), which play a major role in adipocyte differentiation [39], regulate PPARγ, and thereby suppress lipogenesis [40]. Moreover, Okuno *et al*. demonstrated that ω-3 PUFAs also down-regulated genes of the late phase of adipocyte differentiation, such as PPARα and aP2 in rat visceral adipose tissue [27].

We hypothesise that the reduced expression levels of these two genes and other enzymes and transporters important for lipid and glucose metabolism, such as LPL, FAS, CPT-1β and Glut4, are a consequence of DHA inhibiting the two master regulators PPARγ and C/EBPα and thus suppressing adipogenesis and concomitant lipogenesis in C3H10 T1/2 cells. Collectively, our data are both consistent with and extend earlier studies performed in 3T3-L1 cells [41,29,24].

Furthermore a large body of studies concerning β-carotene and lycopene, EGCG and resveratrol demonstrates that the effects of natural compounds on adipocyte differentiation are as varying as their structure [23]. EGCG, the major catechin in green tea, is known to stimulate apoptosis, inhibit adipogenesis and intracellular TG accretion in 3T3-L1 adipocytes [42,43]. Human AML-1 cells undergo apoptosis after treatment with EGCG, although the conversion from pre-adipocytes to adipocytes is not affected [44]. Moreover, EGCG had no lipolytic influence on mature C3H10 T1/2 adipocytes [45]. Our results show that EGCG only moderately inhibited the average intensity of the fat droplets in C3H10 T1/2 cells and it had no significant impact on any gene expression levels, although Furuyashiki *et al*. [43] reported that in 3T3-L1 cells PPARγ and C/EBPα were down-regulated by tea catechins, like EGCG. These also showed an enhancing effect on the expression and secretion of adiponectin in 3T3-L1 adipocytes [46]. However, in maturing C3H10 T1/2 adipocytes the mRNA level of adiponectin was not affected by EGCG. Investigations on the protein level and of the cell supernatants are required to further determine the effects of dietary constituents on secreted adipokines.

Resveratrol has been tested on adipocytes [47] and was found to be apoptotic [48], anti-adipogenic [48,49] and anti-lipogenic [21]. In this study resveratrol showed no inhibitory effect, or even induced a moderate stimulation of lipid accumulation in differentiating C3H10 T1/2 cells. This is consistent with the observed up-regulation of aP2 mRNA at low resveratrol concentrations, compared to rosiglitazone control. In contrast, Rayalam *et al*. [48] reported suppression of adipocyte-specific genes such as PPARγ, C/EBPα, FAS, HSL and LPL and a strong inhibition of lipid accumulation (40% by 25 μM resveratrol) in maturing 3T3-L1 adipocytes. These effects were accounted for by decreased cell viability.

Reduced cell number and increased LDH levels are indicative of apoptosis and reduced cell viability [48]. Consequently, a diminished number of fat cells during differentiation enable less fat accumulation. It can be assumed that β-carotene induced apoptosis at 2 μM similar to EGCG at 25 μM (data not shown, [42]). At this physiologically-relevant concentration β-carotene efficiently reduced fat droplet number, size and intensity. Furthermore β-carotene treatment led to a down-regulation of the dominant regulators of adipogenesis, PPARγ and C/EBPα, and a significant reduction of LPL, HSL, FAS, aP2 and adiponectin. The effects of β-carotene and its biologically active form vitamin A (retinol/retinal/retinoic acid) on WAT are well-studied (for review see [50]). Kawada and co-workers [51] described that fat soluble vitamins, including provitamin β-carotene, strongly inhibited adipose conversion of 3T3-L1 cells. They [52] suggested that carotenoids and retinoids inhibit adipocyte differentiation through retinoic acid receptor up-regulation and the decrease of PPARγ2. The strong suppression of LPL, one of the early markers of adipocyte differentiation [53], is remarkable compared to all the other dose-dependent effects of β-carotene. Insulin is known to induce LPL synthesis in adipocytes and stimulate LPL, ACC and Glut4 proteins. Plausibly, β-carotene - even in low concentrations - interferes with insulin actions, although recently it was shown by Kameji *et al*. [54] that high concentrations of β-carotene elevated LPL and PPARγ levels and insulin functions were supported. Lycopene, as a fat-soluble molecule is also stored in adipose tissue, in particular in different cellular compartments of the adipocytes [55]. Our results illustrate that lycopene decreased the accumulation of TGs to a far lesser extent than β-carotene and it also showed no effect on the different adipogenic and lipogenic genes. Only Glut4, which participates in insulin-dependent glucose uptake into adipocytes [56] and is thus indirectly involved in fatty acid synthesis, was significantly reduced. Therefore, the small effect it has must be mediated via other mechanisms than PPARγ and C/EBPα suppression.

Hydroxytyrosol (HT) is the major polyphenol in extra-virgin olive oil and its potent antioxidant properties are thought to be partly responsible for the Mediterranean diet effect [57,58]. In our study, HT showed effects that were comparable to those of EPA and β-carotene on adipocyte lipid content and gene expression. HT significantly inhibited all tested genes except LPL.

In summary, dietary bioactives such as ω-3 PUFAs, β-carotene and HT, which seem to have the same inhibitory effect on lipid accumulation during adipocyte differentiation, display different impacts on the expression of relevant genes. Therefore, the influence of substances on the phosphorylation status of corresponding enzymes, e.g. LPL and HSL, should also be considered.

The conflicting data reported in different studies might be partly explained by the use of different cell models from different species. Adipose tissue biology is diversely regulated among different species, thus the same might be applicable for different cell lines. Contradictory effects of substances in diverse cell systems could be explained due to the application of cAMP elevating agents, the hormonal status (e.g. dexamethasone and insulin) present during adipocyte differentiation [9] and the stage of the adipocyte life cycle at which they are applied [23].

## Conclusion

Obesity is characterised by increased adipose tissue mass and is associated with high health risks. The expansion of WAT is conditional upon its ability to increase the number of adipocytes and their volume (triglyceride content in fat droplets). Identifying compounds that suppress the formation of new adipocytes and the accretion of fat at different stages of the adipocyte life cycle is of major interest for preventive measures and therapeutic applications. Our data show that this newly developed HCA assay is a valuable tool to identify and quantify changes in fat accumulation during adipogenesis and lipogenesis and thus for the identification of such substances. This study demonstrates that treatment of murine C3H10 T1/2 pre-adipocytes with naturally-occurring components can reduce the amount of accumulated fat. Furthermore, the observed morphological changes are in line with the alterations in expression levels of adipogenic and lipogenic genes.

Murine cell lines might give indications how food ingredients influence the formation of fat droplets in tissues. This needs, however, to be further corroborated by experiments with human adipocytes. Ultimately, studies with obese humans would be of great value, as obesity is not only caused by high fat consumption but also triggered by high intakes of refined carbohydrates [9]. Other research groups approach this challenge and discovered both differences and analogies in human and rodent adipogenesis [59]. For example Söhle *et al*. describes that a white tea extract rich in polyphenols inhibited the adipogenesis in human subcutaneous pre-adipocytes [60].

Because several natural compounds act in different ways on the elaborate biological pathways of adipose tissue formation, it would be advantageous to treat adipocytes with a combination of dietary bioactives. Hence, such an approach might exceed the favourable effects of each individual compound and lead to additive or synergistic effects on multiple levels of adipocyte differentiation. This strategy might also apply for improving the appearance of dimpled skin caused by overfilled subcutaneous adipocytes mostly observed in obese people.

## Competing interests

The authors declare that they have no competing interests.

## Authors' contributions

The author(s) have made the following declaration about their contributions: Conceived and designed the experiments: IW and JS. Performed the experiments: IW and EF. Analysed the data: IW and EF. Contributed reagents/materials/analysis tools: IW, JS and EF. Wrote the paper: IW, RG and JS. All author(s) read and approved the final manuscript.
